# Augmenting the Senses: A Review on Sensor-Based Learning Support

**DOI:** 10.3390/s150204097

**Published:** 2015-02-11

**Authors:** Jan Schneider, Dirk Börner, Peter van Rosmalen, Marcus Specht

**Affiliations:** Welten Institute, Open University of the Netherlands, 177 Valkenburgerweg, Heerlen 6419AT, The Netherlands; E-Mail:dirk.boerner@ou.nl (D.B.); peter.vanrosmalen@ou.nl (P.R.); marcus.specht@ou.nl (M.S.)

**Keywords:** sensors, feedback, sensor-based learning support

## Abstract

In recent years sensor components have been extending classical computer-based support systems in a variety of applications domains (sports, health, *etc*.). In this article we review the use of sensors for the application domain of learning. For that we analyzed 82 sensor-based prototypes exploring their learning support. To study this learning support we classified the prototypes according to the Bloom's taxonomy of learning domains and explored how they can be used to assist on the implementation of formative assessment, paying special attention to their use as feedback tools. The analysis leads to current research foci and gaps in the development of sensor-based learning support systems and concludes with a research agenda based on the findings.

## 1. Introduction

The digital and physical worlds are currently merging, opening new possibilities for us to interact with our environment, as well as for our environment to interact with us. This development is mainly driven by two technologies: display technologies and sensor technologies. Display technologies in the sense of personal mobile displays, as also a variety of embedded public displays, enable the integration and presentation of digital information and services in nearly every situation and context [1]. Sensor technologies enable the development of real-time information systems and the extension of classical objects to be enhanced and integrated into digital eco-systems. Everyday objects, which previously did not seem aware of the environment at all, are turning into smart devices with sensing and tracking capabilities. Cisco estimates that by 2020 there will be 50 billion devices connected to the Internet [2] and one of the main drivers for this to happen is the increasing number of low-cost sensors available [3].

A sensor is commonly defined as: “a device that detects or measures a physical property and records, indicates, or otherwise responds to it.”[4]. The mere linguistic definition of a sensor seems restrictive, in the sense that specific computer programs have been used as sensors, by tracking recent songs played, current URLs open, log of incoming calls and some other non-physical properties [5].Consequently, the definition of a sensor being used in this review is: “a physical or virtual object used for tracking, recording or measuring.” An overview of the identified sensors together with their measured properties and identified usages is shown in Appendix A. Coupling sensors with software components creates new types of tools with the capability to measure, analyze and (immediately) present results of the obtained data. The name for these instruments has not been standardized yet, and in previous works they have been referred as smart-sensors [6] sensor systems [7], sensor platforms [8], ecosystems [3], *etc*. In the remainder of this article these tools will be denoted as sensor-based platforms.

The ability of sensor-based platforms to act according to their retrieved and analyzed data suggests a possible use of them as learning tools. In order to get an overview of the state-of-the-art of sensor-based learning support and to find directions for further research on it, in this literature review we analyzed the learning support of sensor-based platforms that were designed for educational purposes as well as sensor-based platforms that were designed for other purposes but that are also able to support learning through the presentation of relevant information for performance support, analysis and contextual awareness. With the purpose to get an overview of the different areas of learning that have already been influenced by sensor-based platforms, we started our study analyzing the connections between the different types of sensor-based platforms and their support for the commonly distinguished learning domains: the cognitive, psychomotor and affective domain [9]. Since one of the current educational challenges is the implementation of formative assessment [10], within the learning domains we in particularly focused on exploring whether sensor-based platforms can assist on its implementation. Formative assessment provides learners with information that allows them to improve their performance and learning. In our study we carefully analyzed how sensor-based platforms have been used as feedback tools, since formative assessment includes high quality feedback, which should be given as soon as possible after submission; be relevant to the task and the pre-defined assessment criteria; and should help the student to understand how to improve her work (not just highlighting strengths and weaknesses)[11]. However, the required effort for this type of assessment easily leads to a work overload for teachers forcing them to give merely summative instead of formative feedback [12]. Implementing formative assessment with more human work force is currently not a feasible solution, therefore in this review we explored whether sensor-based platforms can contribute to it.

To summarize, this article gives an overview on how sensor-based platforms have been used for learning support, by exploring their contribution on the different learning domains, the implementation of formative assessment, and their status as feedback tools. The remainder of this article is organized as follows: Section 2 presents the classification framework used to analyze the prototypes described in the articles. Section 3 gives an outline of the used methodology. Section 4 presents the results of the analysis. Finally, Section 5 discusses the results and presents an outline for further research on the topic.

## 2. Classification Framework

With the purpose of identifying the already existing best practices for the use of sensors in learning as well as identifying directions for the further development on the state-of-the-art of sensor-based learning support, in this review we examined and studied the current link between learning support and the state-of-the-art of sensor-based platforms prototypes found in literature. In order to conduct our research we proposed a classification framework examining:

- Learning domains: get an overview of sensors and learning.
- Formative assessment: focus our research in sensors and learning, exploring how they can assist with a main current educational challenge.
- Feedback: deepening our research in sensors and learning studying how they have been used for giving feedback, which is a key element for formative assessment and one of the most important interventions in learning.

To get an overview of the type of learning support that has already been tinted by sensor applications, we first analyzed and classified the existing sensor-based platforms according to the support that they give in the commonly identified learning domains [9]. This classification seems suitable because to our knowledge it covers all aspects of learning, allowing us to get an impression of the development of sensor-based learning support, highlighting the areas of learning that have been already influenced by sensors.

The unobtrusive capabilities of sensor-based platforms to measure and analyze data lead us to think of their possible support for assessment. Therefore we deepen our analysis exploring how the state-of-the-art of sensor-based platforms can assist in the implementation of formative assessment, which is a current educational challenge. To study this contribution we analyzed how the state-of-the-art of sensor-based platforms can be used to assist in the 9 aspects of formative assessment that have been identified in [13,14]. Feedback is a key aspect of formative assessment and one of the most important influences in learning [15], hence to gain insight in the effectiveness of sensor-based platforms as feedback tools, we studied their feedback based on the framework of effective feedback [15].

### 2.1. Classification Framework for Learning Domains

**Figure 1.**
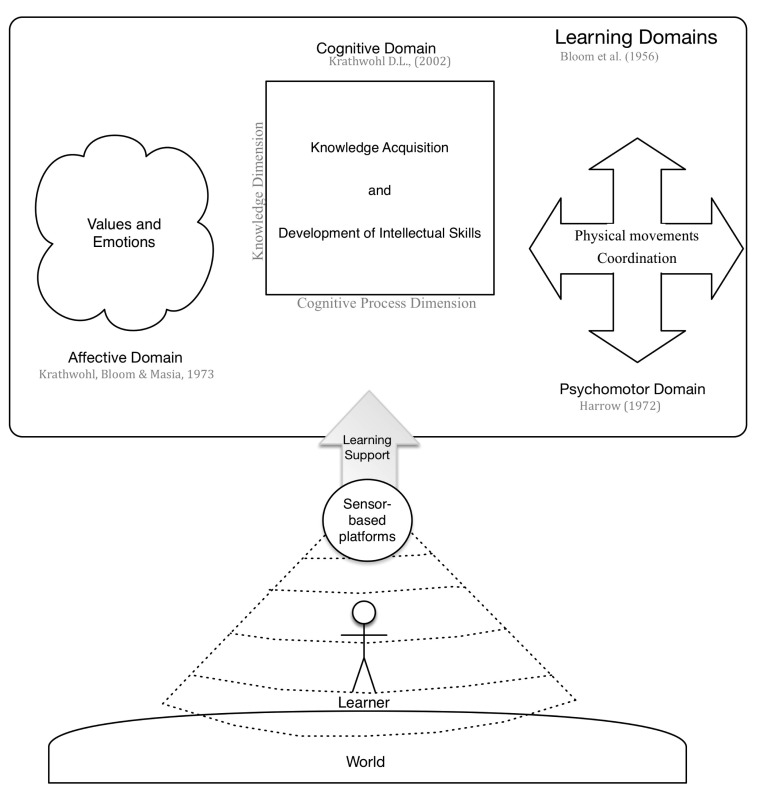
Sensor-based learning support on the learning domains.

Currently most well known sensor applications on the market, such as the Polar heart rate monitors[16], Nike+[17], Digifit[18], or Xbox fitness[19]are used in the field of sports. They are designed to track and give feedback about the physical performance of the users, helping them in training their motoric skills. With the intention to explore whether the use of sensor data can go beyond that, we explored in scientific literature the areas where learning support have been given by sensor-based platforms. For that we analyzed the prototypes described in literature according to their support given on the commonly identified learning domains. These domains are: the cognitive, affective and psychomotor domain[9] (see Figure 1). The cognitive domain refers to knowledge and the development of intellectual skills. It includes the recall or recognition of facts, and the development of intellectual abilities and skills[9]. This learning domain contains two dimensions: the knowledge dimension and the cognitive process dimension. The knowledge dimension refers to the type of knowledge that can be acquired and consists of four categories: factual, conceptual, procedural and metacognitive knowledge. The cognitive process dimension deals with how the knowledge is used. It contains six categories ranging from remembering facts to the creation of new concepts and objects using the acquired knowledge[20].In order to get an understanding on how sensors can support the cognitive domain of learning, we explored the practices that have been used by sensor-based platforms to support these two dimensions.

The affective domain refers to the approach in which learners deal emotionally with things, such as values, feelings, motivations and attitudes. This domain is usually categorized according to the complexity of the behavior incorporated by the learner. Starting from being open to receive the phenomena to internalize these phenomena until they become a characteristic feature of the learner [21]. In this review we explored how the identified prototypes have been used to affectively support learning, enabling us to extract and analyze the strategies used by sensor-based platforms to present support on the affective domain.

The psychomotor domain deals with physical movement, coordination and the use of the motor-skill areas. The development of these skills requires practice and it is evaluated in terms of precision, distance, speed or techniques in execution. Six categories have been identified for this domain: reflex movements, fundamental movements, perceptual, physical activities, skill movements, and non-discursive communication [22]. To explore the current sensor-based learning support on the psychomotor domain of learning, we investigated which of these categories have already been supported by sensor-based platforms and analyzed how this support has been achieved.

### 2.2. Classification for Formative Assessment Support

**Figure 2.**
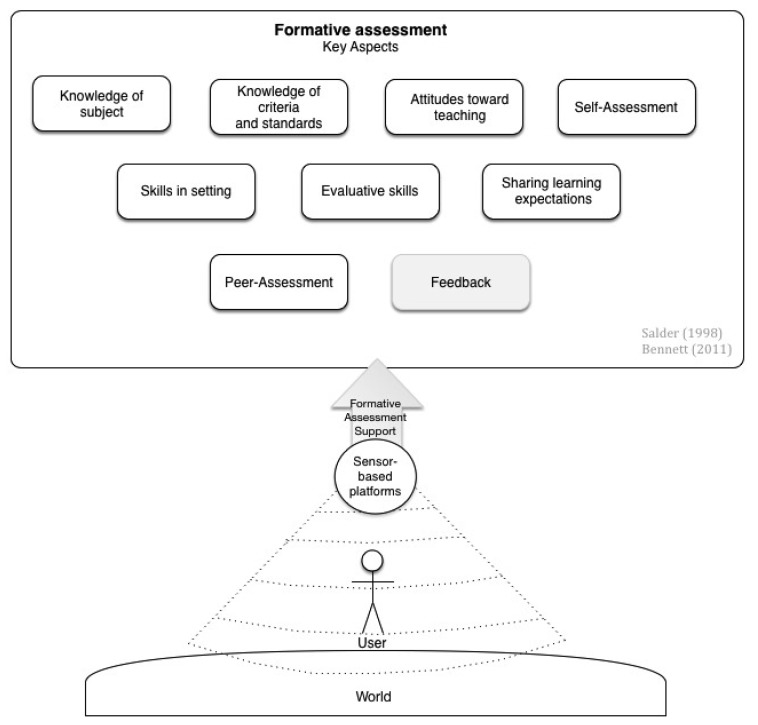
Sensor-based support on formative assessment.

Once having an overview of the possible use of sensors in learning we wanted to explore whether they can be used to help solving a current challenge in education and learning. As introduced above, sensor-based platforms can unobtrusively measure and analyze data, thus suggesting their use in assessment tasks. Therefore, in this second dimension of our classification framework we have classified the analyzed prototypes according to their functions for formative assessment, investigating in which ways sensor-based platforms can contribute to its implementation. From a broad perspective formative assessment refers to the assessment that provides the learner with information, which allows them to enhance their learning and performance [11]. By examining the qualities that allow highly competent tutors to contribute to formative assessment [13], and the strategies discussed on the “Keeping Learning on Track^®^ Program”[14], we have identified nine aspects that contribute to formative assessment (see Figure 2):

- Knowledge of subject matter, allows analyzing the performance of the learner, identifying the origin of its errors.
- Knowledge of criteria and standards, allows giving learners tasks according to their current level.
- Attitudes toward teaching, deals with the empathy from the tutor towards the students and the desire to help students in their development.
- Skills in setting, referring to the capacity of setting assessments that reveal understanding and skills and testing the desired outcomes.
- Evaluative skills, allowing to make appropriate judgments and to deal with the possible responses of the learners.
- Sharing learning expectations, identifying the learners' expectations and allowing sharing them across the peers.
- Self-Assessment, allowing to structure opportunities to take responsibility of own learning.
- Peer-Assessment, allowing to structure opportunities for activating learners instructional resources for each other.
- Feedback, referring to the evaluative information on the positive and negative features of the student's work.

In this dimension of the classification framework we investigated how the sensor-based prototypes described in literature support these aspects of formative assessment. The analysis of feedback, an essential aspect of assessment, will be done separately and discussed in the next section.

### 2.3. Classification Framework for Feedback

Feedback is one of the most powerful interventions in learning [15], and one of the most beneficial thing tutors can do to students is to provide them with feedback that allows them to improve their learning[23]. High quality feedback is a key element of formative assessment [11]. Therefore, we decided to analyze the type of feedback given by the studied prototypes. Feedback in this study is defined as the information about a person's behavior or performance of a task, which is used as a basis for improvement [4]. The effective feedback framework in [15] focuses on how feedback can be used to positively influence the learning process. Consequently, we analyzed the alignment between the feedback of the studied prototypes and this framework.

**Figure 3.**
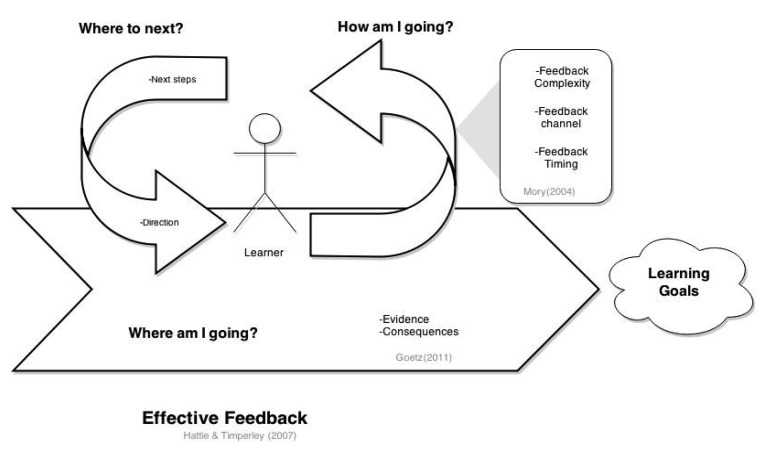
Framework used for the analysis of sensor-based support on effective feedback.

Effective feedback gives answers to the following questions: “where am I going?”, “how am I going?” and “where to next?” (see Figure 3)[15]. The question “where am I going?” refers to the learner's goals; goals produce persistence at task performance while facing obstacles, and support the resumption of disrupted tasks in the presence of more attractive alternatives[24]. The answer to “how am I going?” provides information relative to a task or performance goal of the user. Finally, the answer to “where to next?” shows the learner the next steps to take towards the completion of her goal. Implementing the answers to these questions on a computerized system is not a straightforward task. In order to answer the question of “where am I going?” first it is important to know the goals of the user. The challenge comes in reminding the user about these goals and presenting the user with feedback on how the current task and performance aligns to the goals. Work regarding feedback loops has suggested that by presenting the user with evidence of his current behavior together with the consequences allows the user to perceive an alignment between his performance and goals[25]. Sensors can be used as tools to collect this evidence. Presenting this evidence and the potential consequences is something that can be implemented on a sensor-based platform.

In order to answer “how am I going?”, the performance of the user needs to be tracked, and this performance has to be compared with some rules. The proposed way to classify the type of feedback that gives answer to this second question of is through the five different levels of the complexity of feedback dimension [26], which are:

- No feedback: no indication provided about the performance of the learner.
- Simple verification: indication of correct or incorrect performance of the learner.
- Correct response: indicates the learner how the correct performance should be.
- Elaborated feedback: indicates why the performance of the learner is correct or incorrect.
- Try again feedback: informs the learner when the performance is incorrect and allows her to attempt to change it.

The implementation to the answer of “where to next?” has two basic requirements. First, a map with all steps to achieve the learner's goal is required. Second, it is important to identify the current position of the learner on this map. The measuring and analysis qualities of sensor-based platforms seem suitable to identify the current position of the learner on the learning map. Moreover, sensor-based platforms that make use of system adaptation techniques such as direct guidance, content-based filtering [27], and self-adaptation through feedback loops [28], open the possibility for them to present the learner with a personalized learning map.

In this review, we analyzed how these three questions of effective feedback have been answered by the studied prototypes. To identify the answer to the first question: “where am I going?”,we examined whether the technique described of presenting the evidence together with its consequence [25]has been used by the prototypes, and explored whether some other techniques have been used to address this answer.

For “how am I going?” we analyzed how the feedback given by the prototypes relates to the feedback complexity levels[26].Together with this dimension, we also explored the feedback channel used by the prototypes. This channel can usually be visual, audio or haptic. The reason for this exploration is to investigate whether empirical evidence exists backing up these feedback practices.

For “where to next?” we explored how the prototypes have implemented an answer to this question, presenting attention to the inclusion of system adaptation techniques for personalized answers.

## 3. Method

The purpose of this study is to get an overview on the state-of-the-art of sensor-based learning support and to explore how the existing sensor-based platforms could bring assistance to the solution of an educational challenge, which is the implementation of formative assessment. Therefore we collected articles describing studies about sensor-based prototypes and analyzed them according to our classification framework in order to identify their learning support.

The underlying search for articles was conducted using the online repositories of: Education Resources Information Center Digital Library (ERIC), ScienceDirect (Elsevier),IEEE Computer Society, Association for Computer Machinery and the publisher Springer. The first repository ERIC was selected for being considered the largest repository in education. Elsevier was selected because it contains journals that publish research that merges the technical and educational aspects. The three other repositories were selected for containing the largest digital libraries in computing and engineering.

The search for articles was executed in different phases. The first phase was in the context of an internal study, for which we performed an initial search in early 2013 using the keywords “sensor”, “application” and“learning”. We examined the abstract of these papers looking for computerized applications that have been enhanced by the use of sensors, paying special attention to the ones describing applications that were designed for human learning. This first search left us with 111 articles that were considered relevant for further study.

With the purpose to include the latest research in our repository and to start a formal research on the state-of-the-art of sensor-based learning support, a second search was done in January 2014 using the keywords “sensors”, “software”, “applications” and “learning”, while searching for articles published from 2012 to 2013. The term “software” was added to the query to restrict our search, and to exclude research focused on the hardware of sensors and not on sensor applications. After a scan through the abstracts, looking for applications where sensors have been used for human learning support, 24 articles were selected for a deeper study. While studying the literature we decided to explore more cases where systems have used sensors to adapt their behavior in order to support learners, therefore a later search was performed in March 2014 using the keywords “sensor”, “adaptive”, “system adaptation” and “education” for articles published after 2012. An examination of the abstracts of these search results let us with three articles that have been included in this study.

Finally in order to be sure to include some missing relevant work the state-of-the-art on sensor-based learning support; we included eight more articles and three commercial products to this review that have been pointed out by experts in the field of Technology Enhanced Learning and Human Computer Interaction as representative work in the field of tutoring, feedback and sensor systems.

To select the studies that were included in our analysis, we followed the criteria of including only articles describing sensor-based prototypes, and of which the description of these prototypes presented some information on how they can proportionate some learning support to their users. From the 146 reviewed articles and three commercial products, we were able to identify 112 different sensor-based platforms prototypes. When analyzing articles describing these prototypes, we could identify that only 82 of them include a description of the communication channel between the prototypes and the user. Since this link between the prototype and the user, is the element in a sensor-based prototype responsible to support learning, we decided to only include these 82 prototypes for further analysis.

We conducted the analysis of the prototypes in three stages. On the first stage we explored the learning support given by the prototypes. This support was classified according to the Bloom's taxonomy of learning domains [9] (see Section 2.1). On the second stage we analyzed the contribution of the prototypes in key identified aspects of formative assessment (see Section 2.2). Finally, on the third stage we took a close examination on which of the prototypes did give feedback to the user and how this feedback compared to the effective feedback framework [15] (see Section 2.3).

## 4. Results

Out of the 82 analyzed prototypes that were selected for further analysis, 51 of them were created inside of an educational context specifically designed to support learning; nevertheless by analyzing the description of their communication channel and reports of their usage we identified a total of 79 prototypes providing users with relevant information for evaluation and analysis, performance support or contextual awareness, hence providing users with learning support. We recognized 79 prototypes supporting learning on the learning domains, 51 prototypes contributing to at least one key identified aspect of formative assessment and 35 prototypes giving feedback to the learner. An overview of these prototypes is found in Appendix B.

### 4.1. Classification for Learning Domains

With the intention to get an overview of the learning support that has already been given by sensor applications, we classified the analyzed prototypes according to their support in the different learning domains. Out of the list of 82 prototypes we identified 79 prototypes presenting learning support. By examining the output given by the prototypes, we identified that 56 of them present the user with information that can help her to remember facts, understand concepts, analyze situations, *etc*. Therefore, we classified them as prototypes supporting the cognitive domain of learning. Six of them present information with the purpose to engage users in specific activities, thus we classified them as presenting support to the affective domain of learning. Following this criterion we identified two prototypes supporting both the cognitive and affective domain of learning. The output of 17 of the prototypes presents the learner with information that aims to help her with the improvement of specific movements or her physical abilities. Hence we classified these prototypes as giving support on the psychomotor domain of learning. By analyzing the 56 prototypes that we classified as giving support to the cognitive domain of learning, we could identify three different strategies (see Table 1) that have been used by sensor-based platforms to give this support.

The *first strategy* identified uses sensors to infer the *learner context*, in order to present the learner with relevant contextual information. We identified 22 prototypes following this strategy. The learner's context is commonly inferred by detecting specific objects that are situated in her surroundings. The most common technology that has been used to identify these objects is by attaching Near Field Communication (NFC) or Radio Frequency identification (RFID) tags to them. The sensors of the prototypes are able to read these tags and to present the learner with relevant contextual information. The information presented by the prototypes determines the category of the cognitive domain [20]that receives the learning support. For example, the prototype in [29] presents support on remembering factual knowledge. For this prototype NFC tags have been attached to everyday objects. When the prototype senses one of these tags, information about the tagged object is shown to the learner, this information helps her to remember specific facts about it. The prototype in [30] uses the same strategy. Nevertheless, this prototype supports the category of applying factual knowledge. The purpose of the prototype is to help learners to learn Mandarin, for that it uses GPS sensors to identify the context of the learner and presents him with Mandarin phrases that are suitable to be applied in this context.

The *second strategy* identified on 11 of the prototypes, is similar to the first one; nonetheless this strategy instead of using sensors to track the learner's contexts, it uses sensors to track *specific features* of the learner such as the learning style [31], competences based on the score of predefined pre-tests [32], attention [33–35], emotional state [36,37], uncertainty while using a tutoring system [38,39], trouble solving problems [40] or driving style [41]. The information presented to the learner by these prototypes depends on the tracked values for these features.

The *third strategy* identified uses sensors to gather *relevant data* and presents this data to the learner. We identified 23 of the prototypes following this strategy. For example the prototype of NoiseSpy [42] uses the microphone and GPS of mobile devices to retrieve the amount of noise in different places of a city. These different noise measurements are presented in a map allowing town planners to learn about the noise distribution patterns of a city. In this case the use that the learner gives to this information establishes the cognitive domain category supported by the prototype. This strategy is the only one identified being used by commercial products [43–45]. These products provide different visualizations of sensor data, which could help learners to analyze different phenomena from natural sciences. The application domain for prototypes using this technique of showing sensor data to support the cognitive domain of learning is broad. It can go from the field of civil engineering as in [46], to the field of sports where due to the advances in wearable sensors, human movements are being studied in new and more precise manners [47–49]. Another common application where sensor data supports learning in the cognitive domain is by monitoring the activity, behavior and state of patients in order to gain insight about their health [50–54]. These prototypes have been classified as supporting the cognitive domain of learning instead of the psychomotor domain, because the users of these prototypes who are able to make direct use of the sensor data are experts. By analyzing the data these experts can later use their gained knowledge to give proper advice to patients. This proper advice might indeed support them in the psychomotor learning domain, but it comes from the expert and not from the prototype. The prototype shown in [52] is an example of this; this prototype shows how wearable sensors have been used to monitor the movements of people following a heart stroke helping doctors to select the best therapy for them.

The affective domain of learning deals with attitudes, motivations, values, *etc*. We identified that the information presented to the learner in eight prototypes had the purpose to support them in this domain The analysis of these eight prototypes let us recognize three different strategies that have been used to achieve this support (see Table 2).

The *strategy of behavior overview and review* uses sensors to track certain aspects of the learner's behavior and presents the learner with the overview of it. By doing so, the learner becomes aware of how she is approaching towards the desired goal, motivating her to change or keep up with his current behavior. This strategy has been used by four of the prototypes. The prototype described in [55] exemplifies this strategy. The purpose of this prototype is to engage users into a more active lifestyle, for that, this prototype uses sensors to track the physical activities performed by the user, and displays the overview of them on their mobile devices. Watching the presented activity overview motivates the user to engage into a more active lifestyle.

The *strategy of social network visualization* has been used by two of the prototypes; this strategy lets learners compare themselves with peers of their network, motivating them to perform well in their learning activities. An example of this is described in the prototype in [56]. This prototype presents to students of virtual learning environments some smart indicators informing them about their activities, achievements and progress in comparison with other peer students.

The *strategy of involving learners in data collection* has been identified in two of the prototypes, it supports learning in the affective and the cognitive domain. This strategy has been used to engage learners into scientific activities, by letting them participate in the data-gathering phase of the scientific process. Learners use sensor measurements to gather this data. An example of this strategy is the prototype in [57]. This prototype allows learners to create scientific experiments that are compiled into mobile applications. These applications use the sensors of the mobile devices to assist the learners to conduct their experiments.

Seventeen prototypes have been identified to support the psychomotor domain of learning (see Table 3). For the exploration of this domain we analyzed how the prototypes give support on the six categories of the psychomotor domain of learning [22], identifying support in four of them: *fundamental movements, skilled movements, physical activities* and *non-discursive communication*.

Seven of the prototypes present support to *fundamental movements*, such as walking, running, sitting, *etc*. The purpose of these prototypes is to help patients going through a rehabilitation process. These prototypes use sensors to track the patients' movements, analyze these movements and give feedback to the patients informing them whether the movements have been performed correctly or incorrectly. As an example, the prototype in [58] uses wearable inertial sensors to identify the posture of patients who are going through rehabilitation after a damage of their motor system. Whenever the posture is incorrect the prototype provides audio feedback.

Support for learning *skilled movements*, referring to the movements used for dancing, recreation and sports, has been recognized in seven of the prototypes. The strategy used to support the skilled movements is similar as the one used to support the basic ones, prototypes use sensors to track the learner's movements, analyze how they are being performed and show the analyzed results to the learner. The areas of this type of learning assistance that have been identified are: music gestures [59,60], special rehabilitation exercises [61], taekwondo movements [62], snowboarding [63,64] and karate punches [65].

The prototype in [66] is the only one that has been recognized to support *physical activities*. This prototype uses sensors to track the weather conditions and current fitness of cross-country runners. According to the difficulty of the route, the tracked weather conditions and the tracked current fitness level of the runner, the prototype indicates the runner the route to take for an optimal workout.

Support for learning non-discursive communication referring to the acquisition and development of nonverbal communication skills has been identified in two of the prototypes. The prototype in [67] tracks the facial gestures, voice intonation, volume and speaking rate giving feedback to the learner about the correct use of her nonverbal communication for job interviews. The prototype in [68] is a videogame that tracks the facial expressions in children with autism teaching them how to smile.

### 4.2. Classification for Formative Assessment Support

To explore how sensor-based learning support can contribute to the solution of one current educational challenge [10,11], we studied how the investigated prototypes can bring assistance to the implementation of key aspects of formative assessment (see Section 2.3). By looking at the information that the prototypes gave to the users we identified 51 of them (see Table 4) contributing to at least one of these aspects.

Twelve of the prototypes have been identified to support the aspect of *knowledge of subject matter*, which allows experts on making better assessments about the students' performance. This support is achieved due to the monitoring capabilities of sensors. The sensor data presented to the experts (tutors), helps them to analyze and identify the errors of the learner. This type of support is used in sports and healthcare. An example of the sports field is found in the Swimming prototype [47]. In this prototype, wearable accelerometers are attached to the learner. The data received by these sensors allow for the analysis and error identification of the learner's swimming technique. In healthcare the prototype in [50] uses wearable gyroscopes to analyze the gait of patients. This analysis allows detecting gait abnormalities or deteriorations to identify the presence of diseases and pathologies.

The *knowledge of criteria and standards*, which helps to identify the current learning level of the student, is supported by 16 of the prototypes. The strategy of using sensors to track the learner's performance and to identify his errors, which can be used to support *knowledge of subject matter*, can also be used to identify the current learning level of the learner. Two of the prototypes identify the current level of the learner by identifying his physiological state. The prototype in [34] uses an electroencephalogram to track the attention level of the learner while attending an online lecture. The prototype shows in which part of the lecture the attention of the learner decreases allowing tutors to give tasks to the learner of the subjects in need of being reviewed. The study in [59] describes a prototype that emulates musical sounds according to certain gestures of the users. In this study teachers who observed students using the prototype, reported that the prototype allowed them to identify the musical level of the students.

We identified two prototypes tracking the emotional state of the learner while doing learning tasks and informing the tutor about this [36,37]. This helps the tutor to increase her empathy towards the learner and therefore supports the key aspect of formative assessment identified as *attitudes toward teaching*.

Support for *skills in setting*, which deals with the capacity to set assessments that reveal the knowledge and skills level of students, has been identified in eight prototypes. Four of these prototypes support these aspects by setting assessments to the learners in a spatial context. The prototype in [69] acts as a mobile guide in a museum. It identifies the location of the learner using RFID technology, and according to the location it asks specific questions to the learner and evaluates her answers. Two of the prototypes support *skills in setting* by tracking the physiological state of the learner. These prototypes display this state to the tutor, allowing them to set appropriate assessments according to the learner's identified state. The prototype in [52] exemplifies this. It uses wearable accelerometers to track the movements of patients following a rehabilitation program after having a heart stroke. The analysis of the tracked movements allows doctors to select the right set of exercises and therapy for them. The last identified technique to support skills in setting has been used by two of the prototypes. Here learners are required to use sensors to complete the tests that tutors have given them. For example, in the prototype in [57] students have to collect and analyze data using the sensors of their mobile devices to answer the scientific tests set by the teacher.

Four prototypes support the *evaluative skills*. They achieved this support by evaluating the questions that have been previously asked to the learners. The prototype in [32]has been designed to evaluate the answers of learners to predefined tests and makes use of an expert system to present learners with the learning objects that relate to their tests' results.

Contribution for *self-assessment, i.e.*, structuring opportunities for the student to take responsibility about her own learning, was identified in six prototypes. These prototypes structure opportunities to take responsibility of own learning by showing an overview of the actions and performance of the learner. The prototype in [55]shows an example of this, by tracking the physical activity of the user and displaying an overview of it in the form of a virtual garden where the amount of life displayed in the garden is represented by the physical activity of the user. By looking at this representation, the learner is able to reflect and take responsibility about its actions. Support for key elements such as: sharing learning expectation, and peer assessment have not been identified in the studied prototypes.

### 4.3. Feedback Analysis

Because of its relevance in formative assessment and learning in general, we decided to dedicate a complete subchapter of this review on the analysis of the feedback given by the prototypes. By analyzing the information presented by the prototypes to the user, we could identify that 35 of them, revealed information about the user's performance, activities or states; therefore we selected them for our feedback analysis in this review. In the following subsections of this review we report our exploration on how the questions for effective feedback[15] have been answered by the prototypes.

#### 4.3.1. Where Am I Going?

The answer to “where am I going?” is related to the goals of the user. Five of the prototypes (see Table 5) explicitly display an answer to this question. For example, the user's goal in the prototype described in [70] is to eat healthier and avoid emotional eating. In order to make the user aware of how she stands in respect to her goals, this prototype followed the technique described in [25] of presenting evidence together with consequences. This prototype shows the overview of the user's eating habits as a tree (evidence), where the color (consequence) of the tree depends on the healthiness of the food intake by the user.

The prototypes described in [55,71] used the same technique. The first prototype shows an overview of the healthy activities performed by the user (evidence) as a garden where the amount of flowers and life in the garden (consequence) depend on the amount of physical activities. The second prototype uses the same approach but the metaphor used is the one of an ecosystem. The life of the ecosystem depends on the ecological friendly trips done by the user.

The relevance to answering the question of “where am I going?” by sensor-based platform has been empirically tested in the work in [72]. This work has released two different versions of their prototype. Only one of the versions has presented the user with an overview of her standing in respect to her goal. The results of this study show that the compliance to finish sampling experiences in experience sampling method studies was 23% higher in the group whose participants used the version of the prototype displaying the overview.

#### 4.3.2. How Am I Going?

To answer the question of “how am I going?” the sensor-based platforms are required to track the actions or behaviors of the users, and provide them with information relative to their performance in relation with some predefined rules. Twenty-six of the analyzed prototypes have answered this question (see Table 6). The analysis in this section discusses the *form* and the *channel* of feedback given by the studied prototypes.

*Form of feedback:* Looking at the dimension of complexity of feedback [26], feedback can be given at five different levels including no feedback, simple verification, correct response, elaborated feedback and try again feedback. From the analyzed prototypes one of them gives exclusively a *simple verification* feedback giving the user points when guesses about her glucose levels are correct [51]. Eight of the prototypes present exclusively the “try again” feedback, telling the user that her action was wrong and letting her to repeat the action until it is performed correctly. Six of the prototypes give both the simple verification and the try again feedback. In [73] this has been achieved by playing harmonic sounds when the gait of the users is correct (simple verification) and by playing strong rhythmic sounds pointing out to the user that its gait needs to be corrected (try again feedback).

Ten of the prototypes present elaborate feedback indicating why the performance of the user is correct or incorrect. To give this feedback, the prototypes present the evidence of the user's actions together with indications of the acceptable standards to conduct her activities. An example of this is the prototype described in [62]. This prototype points out the differences between the movements of an expert martial artist (correct technique or correct standard) and the user, letting the user become aware on how to correct her mistakes. The prototype described in [53] used a different feedback strategy, showing that our proposed framework to analyze the feedback of sensor-based platforms to the question of “how am I going?” was not exhaustive. This prototype instead of indicating the user whether her behavior has been correct or incorrect, it presents her with evidence of her tracked behavior and asks her a question about it, presenting her a chance for self-reflection.

Seven studies reported empirical results about the use of the prototype with participants, all of them showing positive results in regards to the purpose of the prototype. Five of these prototypes used the strategy of *try again feedback*[33,60,64,76,80] and two of them presented *elaborate feedback*[67,74].

*Channel of feedback:* Since users receive the feedback through their senses, in theory there is a feedback channel for each one of them: visual, auditory, haptic, gustatory and olfactory. The feedback channels used by the prototypes were audio, visual and a combination of both. Ten of the analyzed prototypes present their feedback exclusively through the audio channel. The prototype developed in [65] has shown an example of this; the sounds played by the prototype depend on the accuracy of the karate punch technique performed by the user. Eight of the prototypes display their feedback through the visual channel. The prototype in [70] uses the screen of the user's mobile device to show a message saying: “let's count slowly to 10 and breath…”. The combination of visual and audio has been used in three prototypes. In [33] the prototype shows the score of the user on the computer screen and plays sounds whenever the user maintains her concentration. Two of the prototypes provided feedback through the haptic channel. The prototype in [60] exemplifies this type of feedback. It consists of a pair of gloves that give haptic feedback when the user, who is learning how to play the violin, performs incorrectly a specific technique.

Empirical positive results in regards to the purpose of the prototype were found for all of the identified feedback channel practices [33,60,64,67,74,76,80]. Pointing out that the study of [64] showed that for physical activities such as snowboarding, the haptic feedback was perceived faster than the audio feedback.

#### 4.3.3. Where to Next?

The answer to “where to next” is about showing ‘some’ guidance to the user on the next steps to follow. Eight prototypes have been identified which present the user an answer to this question (see Table 7) Five of the prototypes present an indicator of just the next step to take, indicating the next step to do to solve a problem [40], showing the steps required to correct mistakes [69], showing the next activity to engage [51], instructing the user the steps that she needs to follow in order for her to relax and gain self-control again during highly emotional situations [70], and showing which direction to take [66]. Three of the prototypes present the user with a complete personalized learning path for them. This path has been obtained by capturing the user's attention levels during a virtual lecture [34], tracking the user's competences [32], or identifying the user's learning styles [31]. While the prototype in[34] has just pointed out the user the steps to follow, the prototypes described in[31,32] have used system adaptation techniques to present the user with her personalized path. The system adaptation technique presented by [31] uses a literature-based approach, where the number of visits and time spend by the students working with learning objects is used to automatically identify the student's learning style. This approach tracks the behavior of students in order to get hints about their learning style preferences, then it uses a rule-based approach to estimate the preferred learning style from the amount of matching hints. Finally, it presents the learner with a learning path suited for his learning style. None of the prototypes have shown empirical results about their learning support.

## 5. Discussion

The pairing of sensors with software components has created tools with capabilities to automatically retrieve and analyze data, referred to in this review as sensor-based platforms. In order to explore the use of these tools in learning, we analyzed the prototypes described in literature according to our classification framework. Starting with an exploration of the areas of learning that have been supported by sensor-based prototypes, this review revealed that sensor-based platforms have been designed and used to give support in each of the three learning domains. The domain with the most support (56 of the 82 studies) is the cognitive domain; mirroring what happens with learning in general, where the cognitive domain is the most used and studied [81]. Remarkably, also given the research in [82], which asserts that a comprehensive educational design should merge these domains, in this review we could only identify two prototypes supporting a combination of domains. This presents a research opportunity on finding the implications to create sensor-based platforms able to support multiple domains of learning.

In our search to seek whether sensor-based platforms can be used to help solving current educational challenges, we continued our analysis of the prototypes studying their possible connection with formative assessment. While in this review we did not identified prototypes specifically designed to give formative assessment, our analysis showed that sensor-based platforms have already been used for seven of the nine aspects of formative assessment described in Section 2.2 (see Table 4). The missing aspects for contribution were structuring opportunities for peer assessment and sharing learning expectations.

With the intention to deepen our research in an aspect considered to be fundamental for learning and for formative assessment, our analysis of the prototypes showed that sensor-based platforms are able to retrieve, measure and analyze personal information in order to give feedback on the three questions of effective feedback. For giving an answer to the first question, “Where am I going?” which deals with guiding learners towards their goal, we identified three different used representations. These representations consisted of a description of the learners' goals, showing the learners' performance together with the consequences, and showing a metaphor of the goals and performance instead of the real data values. While we recognize ways to give an answer to this question, we did not found prototypes attempting to formulate an advice on it. Additionally, none of the reviewed articles studied the appropriate timing for giving this type of feedback. An important aspect for answering the question of “where am I going?” which besides relating to the metacognitive skills of a self-regulated learner one of its main purposes is to keep the learner motivated, therefore having an impact on the affective domain of learning. As previously seen in the analysis of the learning domains, the affective domain of learning does not receive as much attention as the cognitive domain, which partly can explain the knowledge gaps on how to use sensor-based platforms to answer “where am I going?”.

Continuing with the second question, this review shows that sensor-based platforms have been used to give an answer to “how am I going?”. We recognized several feedback representations used by sensor-based platforms to answer this second question. These representations can be classified according to different feedback dimensions [26]such as: timing of feedback, feedback channel and complexity of feedback. However, what we miss from the reviewed articles was a study revealing a suitable method to present this answer as feedback to the learner. From the reviewed articles only [60,64,73]presents an explanation for the selection of its feedback method. Overall, also in relation to the other two questions discussed, studies about the effectiveness of the different feedback channels and feedback dimensions are limited, finding only one work [64] comparing the receptivity between the auditory and the haptic feedback channel. Moreover, no study has been identified exploring how the different ways to give feedback using sensor-based platforms, play a role in subjects such as the cognitive load [83], reflection-in-action and reflection-on-action of the learner [84].

The review shows that sensor-based platforms can be used to show the users their next learning steps, therefore answering the question of “where to next?”. What we miss to recognize in the literature is a prototype able to answer the three questions of effective feedback.

This analysis allowed us to identify two main research branches for sensors-based learning support. The first branch deals with the acquisition of relevant data that might be useful for the learner, and the second branch deals with the presentation of this sensor data to the learner. The amount of different prototypes supporting learning for so many different subjects and domains has shown us that several researches have already been undertaken on the acquisition of relevant sensor data for learning. However, we did not identify many studies investigating and reporting on the implications to deliver this relevant inferred sensor data in ways that can effectively support learning. Looking that only 35 out of 82 prototypes have been identified to present the learner with feedback reveals this. Furthermore, we found only one study [64] analyzing different types of feedback methods for their prototypes, and only in few cases the selection of the feedback methods used by the prototypes were argued. This research gaps give us an indication of the state-of-the-art of sensor-based learning support, which can be corroborated by the very few empirical studies found investigating the effectiveness of sensor-based platforms as learning tools. This current state of research in sensors and learning is also reflected in related literature reviews studying the topic of sensors, where the purpose is to analyze these platforms based on techniques to identify objects [85], achieve ambient intelligence [86], augment reality [87], create body sensor networks [88], classify postures and movements using wearable sensors [89], *etc*. None of the literature studies known to the authors focus on the use of sensors to support learning. These findings about the current maturity of sensor-based learning support align with the lack of use of sensor-applications for formal learning, which are not that popular yet and only deal with the presentation of sensor data for the study natural sciences [43–45] together with the arrival to the market of sensor applications such as Nike+[17], Digifit[18], Xbox fitness[19]*etc*. that support informal learning.

## 6. Conclusions

In this review we analyzed 82 prototypes found in literature studies according to our classification framework in order to identify the state-of-the-art of sensor-based learning support. The analysis revealed sensor-based learning support as an emerging and promising field of study, which has the potential to support learning in several areas and subjects. In this review we merely identified research studies focusing on the learning aspects of the described sensor-based platforms. This turned out to be a limitation for this review, by not allowing us to clearly identify and analyze the learning strategies used by the prototypes. Nevertheless, this lack of focus on learning effectiveness, points out a research direction for further improvement on the state-of-the-art of sensor-based learning support.

This review shows that the focus on sensor-based applications for learning support is quite broad and that this support can have an effect on all the learning domains. It also shows the potential for sensor-based platforms to contribute on the implementation of formative assessment. Nevertheless, we found a lack of studies focusing on the implications required for sensor-based platforms to present their inferred information in such ways that learners can assimilate it effectively, so that sensor-based platforms can become effective learning tools. This research gap suggests the main research path to follow for the improvement of sensor-based learning support. By following this path, we consider that sensor-based platforms can become reliable learning tools able to reduce the workload of human teachers and therefore contribute to the solution of a current educational challenge, which is the implementation of formative assessment. While more work needs to be done on sensor-based platforms to become common learning tools introduced to formal and non-formal learning programs, this review can be taken as a basis and inspiration towards this goal.

## Figures and Tables

**Table 1.** Strategies supporting learning in the cognitive domain.

**Sensor Usage (Design)**	**Number of Prototypes**	**Example of Sensors Used**	**Cognitive Domain Category**
Contextual information acquisition for filtering	22	NFC, RFID, GPS, Microphones	Depends on the information attached to the context
Learner's feature identification and user modeling	11	EEG, Software sensors, NFC, Cameras, Heart-rate monitor	Depends on the information attached to the feature
Sensor Data for contextual reflection and change notification	23	Accelerometers, Air pollutants sensors Cameras, ECG, EEG, gyroscopes, microphones	Depends on the use of the information by the learner

**Table 2.** Strategies supporting learning in the affective domain.

**Strategy**	**Number of Prototypes**	**Example of Sensors Used**
Behavior overview and review	4	Accelerometers, Barometer, Camera, Compass, GPS, Humistor, Microphone Software sensors, Thermometer
Social network visualization	2	Blood glucose meter,Software sensors
Involving learners in data collection	2	Accelerometers, Camera, Microphone, Thermometers

**Table 3.** Overview of the support for learning in the psychomotor domain.

**Category Supported**	**Amount of Prototypes**	**Example of Sensors Used**
Reflex movements	0	-
Fundamental movements	7	Accelerometers, Cameras, ECG, Electromyography sensor, Gyroscopes
Perceptual	0	-
Physical activities	1	Heart-rate monitor, Thermometer
Skilled movements	7	Accelerometers, Cameras, Force gauge, Gyroscopes
Non-discursive communication	2	-

**Table 4.** Support for the aspects of formative assessment.

**Aspects of Formative Assessment**	**Number of Prototypes**	**Strategies Used**	**Example of Sensors Used**
Knowledge of subject matter	12	Presenting sensor data about the learner's performance	Accelerometers,Cameras, Gyroscopes, Software sensors

Knowledge of criteria and standards	15	Presenting sensor data about the learner's performance.	Accelerometers,Cameras, EEG, Heart-rate monitors, Galvanic skin response sensor,Gyroscopes, Software sensors
		Presenting sensor data about the learner's physiological state	

Attitudes toward teaching	2	Informing the tutor about the emotional state of the learner while performing learning tasks	Camera, Galvanic skin conductance, pressure mouse, accelerometers

Skills in setting	8	Setting assessments according to learner's location.	GPS, EEG, Heart-rate monitors,NFC, RFID, Software Sensors
		Setting assessments according to learner's physiological state	

Evaluative skills	4	Evaluating answers of learners	GPS, NFC, RFID, Software Sensors

Sharing learning expectations	0	-	-

Self-Assessment	6	Presenting an overview of the learner's performance	Accelerometers, GPS, Software sensors

Peer-Assessment	0	-	-

Feedback	35	Presenting information about the learner's performance, behavior or state	Accelerometers,Cameras, EEG, Heart-rate monitors,Galvanic skin response sensor,Gyroscopes, Software sensors

**Table 5.** Prototypes answering to “where am I going?”.

**Prototype**	**Topic**	**Strategy Used to Answer the Question**
Carroll *et al.*, (2013)[70]	Healthy eating	Evidence: Overview of eating habits represented as a tree.Consequences: The color of the tree changes.
Consolvo *et al.*, (2008)[55]	Healthy living	Evidence: Overview user's activities represented as a garden. Consequences: Life in the garden depends on the activities.
Froehlich *et al.*, (2009)[71]	Eco-traveling	Evidence: Overview of means of transportation as an ecosystem. Consequences: Life in the ecosystem depends on the means.
Hicks *et al.*, (2010)[53]	Healthy habits	Ask questions about performed activities to reflect about goals.
Hsieh *et al.*, (2008)[72]	Physical activities	Evidence: Overview of user's performance presented together with the goals.

**Table 6.** Prototypes answering to “how am I going?”.

**Prototype**	**Topic**	**Strategy Used to Answer the Question**	**Channel of Feedback**
Aukee *et al.*, (2004) [74]	Incontinence	Elaborate feedback	Visual

Baca & Kornfeind (2006)[75]-Biathlon	Rifle movements in Biathlon	Elaborate feedback	Visual

Baca & Kornfeind (2006)[75]-Rowing	Exerted forces in rowing	Elaborate feedback	Visual

Baca & Kornfeind (2006)[75]-Table tennis	Shot position and cadence in table tennis	Elaborate feedback	Visual

Bevilacqua *et al.*, (2007) [59]	Musical level	Try Again	Audio
		Simple verification	

Brunelli *et al.*, (2006)[58]	Posture	Try Again	Audio

Burish, & Jenkins(1992) [76]	Relaxation	Try Again	Audio
		Simple verification	

Carroll *et al.*, (2013)[70]	Healthy eating	Elaborate feedback	Visual

Cockburn *et al.*, (2008)[68]	Teaching to smile	Try Again	Visual
		Simple verification	

Hicks *et al.*, (2010)[53]	Healthy habits	Questions are asked user letting the user reflect about the answer.	Visual

Hoque *et al.*, (2013)[67]	Interview coaching	Elaborate feedback	Visual

Kranz *et al.*,(2006)[61]	Physiotherapy	Try Again	Audio
			Visual

Kwon & Gross (2005)[62]	Martial arts	Elaborate feedback	Visual

Lehrer *et al.*, (2000)[77]	Breathing technique	Try Again	Audio

Li *et al.*, (2012)[78]	Coordination training	Try Again	Audio
		Simple verification	Visual

Linden *et al.*, (1996)[33]	Attention level	Try Again	Audio
			Visual

Paradiso *et al.*, (2004)[73]	Gait	Try Again	Audio
		Simple verification	

Pentland (2004)[51]Diabetes	Diabetes	Simple verification	Audio

Spelmezan & Borchers (2008)[63]	Snowboarding	Try Again	Audio

Spelmezan *et al.*, (2009)[64]	Snowboarding	Try Again	Haptic

Strachan (2005)[79]	Sound navigation	Try Again	Audio

Takahata *et al.*, (2004)[65]	Martial arts	Try Again	Audio
		Simple verification	

Vales-Alonso *et al.*, (2010)[66]	Cross country running	Elaborate feedback	Visual

Van der Linden *et al.*, (2011)[60]	Violin Playing	Try Again	Haptic

Verhoeff *et al.*, 2009[80]	Gait	Elaborate feedback	Audio

Verpoorten *et al.*, 2009[56]	Indicators for virtual learning environments	Elaborate feedback	Visual

**Table 7.** Prototypes answering to “where to next?”.

**Prototype**	**Strategy Used to Answer the Question**
Anderson & Reiser (1985)[40]	Informs the user which next step to take.
Carroll *et al.*, (2013)[70]	Informs the user which next step to take.
Chen & Huang (2012)[69]	Presents a corrective step to follow.
Dung & Florea (2012)[31]	Presents a personalized learning path.
Hsu & Ho (2012)[32]	Presents a personalized learning path.
Pentland (2004)[51]Memory glasses	Informs the user which next step to take.
Szafir & Mutlu (2013)[34]	Points out the steps to follow.
Vales-Alonso *et al.*, (2010)[66]	Tells the user which direction to take.

## Appendix A

**Table:** Identified Sensors Together with Their Measured Property and Identified Functions

**Sensor**	**Measured Property**	**Helps with**	**Installation**
Accelerometer	Acceleration	Activity sensing, Context sensing, Environment sensing, Physiological state sensing	Environmental, Wearable
Air pollutants sensors	Amount of toxic particles in the atmosphere	Context sensing, Environment sensing	Environmental, Wearable
Barometer	Pressure	Activity sensing, Context sensing, Physiological state sensing	Environmental, Wearable
Blood glucose meter	Glucose on the blood	Physiological state sensing	Wearable
Bluetooth	Radio signals	Activity sensing, Context sensing	Wearable
Camera	Visual light	Activity sensing, Context sensing, Environment sensing, Physiological state sensing	Environmental, Wearable
Compass	Earth magnetic field	Activity sensing	Wearable
Electro cardiogram (ECG or EKG)	Heartbeat	Activity sensing	Wearable
Electrodermal activity meter (EDA)	Skin conductance	Physiological state sensing	Wearable
Electroencephalogram (EEG)	Electrical activity along the scalp	Activity sensing, Context sensing Physiological state sensing	Wearable
Electromyography sensor	Electrical activity produced by skeletal muscles	Activity sensing	Wearable
Force gauge	Force	Activity sensing	Wearable
Galvanic skin response sensor	Skin conductance	Context sensing, Physiological state,	Wearable
Global positioning system (GPS)	Earth coordinates	Activity sensing, Context sensing, Environment sensing	Environmental, Wearable
Global system for mobile (GSM)	Radio signals	Context sensing	Environmental, Wearable
Gyroscope	Measures orientation	Activity sensing, Context sensing, Physiological state sensing	Wearable
Humistor	Detects humidity	Activity sensing, Physiological state sensing	Wearable
Infra red camera	Infra red frequency of light	Activity sensing, Context sensing	Environmental, Wearable
Microphone	Sound waves	Activity sensing, Context sensing, Environment sensing	Environmental, Wearable
Near Field Communication receiver	Radio frequency	Context sensing	Environmental, Wearable
Radio frequency identification receiver	Radio frequency	Context sensing	Wearable,
Sonar	Detect objects through sound waves	Activity sensing	Wearable
Software Sensors	Detect user's actions	Activity sensing Context sensing	Environmental, Wearable
WiFi	Radio frequency	Context sensing	Environmental, Wearable

## Appendix B

**Table:** List of the Analyzed Prototypes

**Prototype**	**Learning Domain**	**Formative Assessment Contribution**	**Sensors Used**	**Description**
Ailisto *et al*., (2006) [90]	Cognitive	-	CamerasRFID readers	It reads tags placed in objects in order to present more information about them.

Anderson & Reiser (1985) [40]	Cognitive	Feedback	Software Sensors	A tutoring system that helps students when having trouble solving some problems for the lisp programing language.

Amaratunga *et al*., (2002) [46]	Cognitive	-	Accelerometers	It monitors the movement of a flagpole and streams this data through the network creating a virtual lab.

Arroyo *et al*., (2009) [36]	Cognitive	Attitudes towards teaching	Camera, Galvanic skin conductance, pressure mouse, accelerometers.	It is a prototype that detects the emotional state of students while interacting with an intelligent tutoring system.

Baca & Kornfeind (2006) [75]-Biathlon	Cognitive	Knowledge of subject matter	Camera	It analyzes the movement of the rifle.
		Knowledge of criteria and standards		
		Feedback		

Baca & Kornfeind (2006) [75]-Rowing	Cognitive	Knowledge of subject matter	Force transducer	It is analyzes the rowing technique.
		Knowledge of criteria and standards		
		Feedback		

Baca, & Kornfeind (2006) [75]-Table tennis	Cognitive	Knowledge of subject matter	Accelerometers	It analyzes the position of the table tennis shots.
		Knowledge of criteria and standards		
		Feedback		

Börner *et al*., (2014) [35]	Cognitive	-	Camera	It is a ambient display that adapts its behavior to capture the attention of learners.

Broll *et al*., (2011) [91]	Cognitive	-	NFC	It is a game where players need to touch parts of a screen with NFC readers

Chapel (2008) [92]	Cognitive	-	GPS	It provides a communication system for students in a university.
			NFC	
			WiFi	

Chavira *et al*., (2007) [93]	Cognitive	-	RFID	It gives contextual information to the participants of a conference.
			NFC	

Chen & Huang, (2012) [69]	Cognitive	Skills in setting	RFID	It gives a tour through a museum.
		Evaluative skills		
		Feedback		

Chu *et al*., (2010) [94]	Cognitive	-	RFID	It gives a tour through a botanic garden.

Dung & Florea (2012) [31]	Cognitive	Feedback	Software sensors	It detects the learning style of students and presents them later with learning objects fitting their style.

Edge *et al*., (2011) [30]	Cognitive	-	GPS	It helps to learn a second language by presenting users with contextual phrases.

Ghasemzadeh *et al*., (2009) [48]	Cognitive	Knowledge of subject matter	Accelerometers	It analyzes golf swings.
		Knowledge of criteria and standards		

GlobiLab for middle & high schools [43]	Cognitive	-	-	Commercial software to visualize and analyze sensor data.

Greene (2010) [50]	Cognitive	Knowledge of subject matter	Gyroscopes	It analyzes the user's gait.
		Knowledge of criteria and standards		

Hester *et al*., (2006) [52]	Cognitive	Knowledge of criteria and standards	Accelerometers	It measures the movements of people who have suffered a heart stroke.
		Skills in setting		

Hicks *et al*., (2010) [53]	Cognitive	Self-Assessment	Accelerometer	It captures the health and behavior of the user, with the sensors of a mobile device
		Feedback	GPS	

Hsu & Ho (2012) [32]	Cognitive	Knowledge of subject matter	NFC	It chooses the learning path of the learner according to its tracked competences
		Knowledge of criteria and standards		
		Evaluative skills		
		Feedback		

James *et al*., (2004)[47] swimming	Cognitive	Knowledge of subject matter	Accelerometers	It analyzes the movements of the user while swimming.
		Knowledge of criteria and standards		

James *et al*., (2004) [47] rowing	Cognitive	Knowledge of subject matter	Accelerometers	It analyzes the movements and applied forces of the user while rowing.
			GPS	
		Knowledge of criteria and standards		
			Heart rate monitors	

Jraidi, I., & Frasson, C. (2012) [38]	Cognitive	-	EEG	It detects the uncertainty of students while performing exercises in an intelligent tutoring system.

Kaasinen *et al*., (2009) [29]	Cognitive	-	NFC	It reads tags placed in objects in order to present more information

Kanjo(2009) [42] MobAsthma	Cognitive	**-**	Air pollutants sensors	It measures the air pollution and compares it asthma cases.
			GPS	
			Software sensors	

Kanjo(2009) [42] NoiseSpy	Cognitive	**-**	GPS	It measures the noise pollution.
			Microphones	

Kanjo(2009) [42] PollutionSpy	Cognitive	**-**	Air pollutants sensors	It measures the air pollution.
			GPS	

Karime *et al*., (2011) [95]	Cognitive	-	RFID	It is a magic wand that recognizes objects and displays information about them on an ambient screen.

Kozaki *et al*., (2010) [96]	Cognitive	Knowledge of subject matter	Accelerometers	It tracks the motion of the user in order to infer its activity
		Knowledge of criteria and standards	ECG	

Kubicki *et al*., (2011) [97]	Cognitive	-	RFID	It is an interactive tabletop able to identify tangible objects.

Kuflik *et al*., (2011) [98]	Cognitive	-	RFID	It is a mobile guide for museums.

Lee & Carlisle (2011) [54]	Cognitive	Knowledge of subject matter	Accelerometers	It detects falls using the accelerometer of mobile devices.
		Knowledge of criteria and standards	GPS	

Linden *et al*., (1996) [33]	Cognitive	Feedback	EEG	It trains children with ADD to pay attention.

Littlewort *et al*., (2011) [37]	Cognitive	Attitudes towards teaching	Camera	This prototype tracks the facial expressions of kids while solving problems using a tutoring system.

Logger Pro [44]	Cognitive	-		Commercial Software to visualize sensor data.

Lu *et al*., (2009) [99]	Cognitive	-	Microphone	It uses the microphone of the mobile device of the user to identify its context and present information about it.

Mandula *et al*., (2011) [100]	Cognitive	Skills in setting	RFID	It identifies indoor locations and objects in order to give contextual information to the user.
		Evaluative skills		

Maisonneuve *et al*., (2009) [101]	Cognitive	-	Microphone	It measures the noise pollution.
			GPS	

Muñoz-Organero *et al*., (2010) [102]	Cognitive	-	RFID	It uses sensors to identify objects and give information about them.

Muñoz-Organero *et al*., (2010) [103] Network Lab	Cognitive	Skills in setting	RFID	It identifies objects on a network lab and gives information about them to the users.

Nijholt *et al*., (2007) [104]	Cognitive	-	Cameras	It captures and displays the non-verbal input of the user.

Ogata *et al*., (2006) [105]	Cognitive	-	GPS	It helps to learn a second language by presenting users with contextual phrases.
			RFID	

Pentland (2004) [51] Medical Monitoring	Cognitive	Knowledge of subject matter	Accelerometer	It monitors the health condition of the user.
			Blood pressure sensor, EEG	
		Knowledge of criteria and standards		
			Heart-rate monitor	
			Galvanic skin sensor GPS	
			Thermometers	

Pentland (2004) [51] Memory Glasses	Cognitive	Feedback	Bluetooth	It triggers reminders to the user according to its context
			GPS	
			Software Sensors	

Pérez-Sanagustín *et al*., (2012) [106]	Cognitive	-	NFC	It provides contextual information to students inside of the university campus.
			RFID	

Rahman, & El Saddik (2012) [107]	Cognitive	-	Accelerometers	It gets information about objects by pointing at them.
			Infrared cameras	

Ramirez-Gonzalez *et al*.,(2012) [108]	Cognitive	Skills in setting	NFC	It allows teachers to create information about objects so that students can access this information later.

Serbedzija & Fairclough (2012) [41]	Cognitive	-	Heart-rate monitor	It adapts the cockpit of a car according to the user's state and driving rules.
			Electromyography	
			GPS Speedometer	

SPARKvue [45]	Cognitive	-		Commercial Software to visualize sensor data.

Spelmezan, Schanowski & Borchers (2009) [49]	Cognitive	-	Bend sensors	It tracks important moments during snowboarding.
			Force sensors	
			Inertial sensors	
			Software Compass	

Strachan (2005) [79]	Cognitive	Feedback	GPS	It helps users to navigate through sounds.

Szafir & Mutlu (2013) [34]	Cognitive	Knowledge of criteria and standards	EEG	It tracks the attention level of students during a virtual lecture, and recommends which subjects to review after it.
		Skills in setting		
		Feedback		

Whitehill *et al*., (2008) [39]	Cognitive	Knowledge of criteria and standards	Camera	The prototype determines the speed at which lesson material should be presented in a tutoring system according to the facial expressions of the learner.

Garrido (2011) [109]	Cognitive - Affective	Skills in setting	NFC	It is a game where objects can be found by using sensors.
		Evaluative skills	RFID	

Heggen (2012) [57]	Cognitive - Affective	Skills in setting	Accelerometer	It uses the sensors on a mobile device to gather scientific data.
			Camera	
			Microphone	
			GPS	

Carroll *et al*.,(2013) [70]	Affective	Self-Assessment	Accelerometer	It monitors the emotional state of the user and keeps track of its eating habits.
			ECG	
		Feedback	Electro dermal activity	

Consolvo *et al*., (2008) [55]	Affective	Self-Assessment	Accelerometer Barometer	It monitors and keeps track of the physical activity of the user.
			Camera	
		Feedback		
			Compas	
			Humistor	
			Microphone	
			Thermometer microphone	

Froehlich *et al*., (2009) [71]	Affective	Self-Assessment	Accelerometer	It monitors and keeps track of the means of travel by the user.
			Barometer	
		Feedback	Infrared camera	
			GSM	

Hsieh *et al*., (2008) [72]	Affective	Self-Assessment	Software sensors	It monitors and keeps track of the user's activities.
		Feedback		

Pentland(2004) [51] DiabeNet	Affective	Self-Assessment	Blood glucose meter	It monitors the glucose condition of the user.
			Software sensors	
		Feedback		

Verpoorten *et al*., (2009) [56]	Affective	Feedback	Software sensors	It adapts a virtual learning environment according to the learner's actions and interests.

Aukee *et al*., (2004) [74]	Psychomotor	Feedback	Biofeedback(Barometer)	It gives feedback about the pelvic floor activity, and it is used to improve incontinence.

Bevilacqua *et al*., (2007) [59]	Psychomotor	Knowledge of criteria and standards	Accelerometers	It maps gestures taught on a music lesson to sounds.
		Feedback	Gyroscopes	

Brunelli, *et al*., (2006) [58]	Psychomotor	Feedback	Accelerometers	It corrects the posture of people going through a rehabilitation process.
			Inertial sensors	

Burish, & Jenkins (1992) [76]	Psychomotor	Feedback	Electromyograph	It teaches patients going through Chemotherapy how to relax.
			Thermometer	

Cockburn *et al*., (2008) [68]	Psychomotor	Feedback	Cameras	It is a game that trains children with autism to perform some facial gestures.

Hoque *et al*., (2013) [67]	Psychomotor	Feedback	Cameras	It is a prototype that helps learners to develop social skills for job interviews
			Microphones	

Kranz *et al*., (2006) [61]	Psychomotor	Feedback	Accelerometers	It corrects the movements of patients going through physiotherapy.
			Gyroscopes	
			RFID	

Kwon & Gross (2005) [62]	Psychomotor	Feedback	Accelerometers	It is a motion training system for martial arts.
			Cameras	

Lehrer *et al*., (2000) [77]	Psychomotor	Feedback	ECG	It trains users to breath according to the heartbeat.

Li *et al*., (2012) [78]	Psychomotor	Feedback	Camera	It is a game based psychomotor skill training for kids with autism.

Paradiso *et al*., (2004) [73]	Psychomotor	Feedback	Accelerometers	It produces different sounds according to the gait of the users.
			Barometers	
			Gyroscopes	
			Sonar	

Spelmezan & Borchers (2008) [63]	Psychomotor	Knowledge of subject matter	Bend sensors	It helps to train the snowboarding technique.
			Force sensors	
		Knowledge of criteria and standards	Inertial sensors	
		Feedback	Software Compass	

Spelmezan *et al*., (2009) [64]	Psyhomotor	Feedback	Bend sensors	It helps to train the snowboarding technique using haptic feedback.
			Force sensors	
			Inertial sensors	
			Software Compass	

Takahata *et al*., (2004) [65]	Psychomotor	Feedback	Accelerometers	It helps to train karate movements.
			Cameras	

Vales-Alonso *et al*.,(2010) [66]	Psychomotor	Feedback	Barometer	It helps cross country runners with its training.
			Heart-rate monitor	
			Humistor	
			Thermometer	

Van der Linden *et al*., (2011) [60]	Psychomotor	Feedback	Inertial motion capture sensors	It is a prototype that helps learners to practice certain movements while playing violin.

Verhoeff *et al*., (2009) [80]	Psychomotor	Feedback	Accelerometers	It gives feedback according to the user's gait.
			Gyroscopes	

Chang *et al*., (2009) [110]	-	-	Humistor	It is a house that adapts certain aspects automatically according to the user's preferences
			Light sensors	
			NFC	
			RFID	
			Thermometers	

Hsu (2010) [111]	-	-	RFID	It is a house that adapts the music being played automatically according to the user's preferences.

Krause *et al*., (2006) [112]	-	-	Accelerometer	It is a mobile phone that changes its behavior according to the user state and surroundings.
			Galvanic skin response	
			Thermometer	
